# miR-21 in Human Cardiomyopathies

**DOI:** 10.3389/fcvm.2021.767064

**Published:** 2021-10-27

**Authors:** Surina, Rosaria Anna Fontanella, Lucia Scisciola, Raffaele Marfella, Giuseppe Paolisso, Michelangela Barbieri

**Affiliations:** ^1^Department of Advanced Medical and Surgical Sciences, University of Campania “Luigi Vanvitelli”, Naples, Italy; ^2^Mediterrannea Cardiocentro, Napoli, Italy

**Keywords:** miR-21, cardiomyopathies, biomarkers, targeted therapy, fibrosis

## Abstract

miR-21 is a 22-nucleotide long microRNA that matches target mRNAs in a complementary base pairing fashion and regulates gene expression by repressing or degrading target mRNAs. miR-21 is involved in various cardiomyopathies, including heart failure, dilated cardiomyopathy, myocardial infarction, and diabetic cardiomyopathy. Expression levels of miR-21 notably change in both heart and circulation and provide cardiac protection after heart injury. In the meantime, miR-21 also tightly links to cardiac dysfunctions such as cardiac hypertrophy and fibrosis. This review focuses on the miR-21 expression pattern and its functions in diseased-heart and further discusses the feasibility of miR-21 as a biomarker and therapeutic target in cardiomyopathies.

## Introduction

Cardiomyopathy refers to heart muscle diseases that show functional and structural myocardial abnormalities such as increasing thickness, dilation, and stiffness (1). As a result, the heart muscle is weakened, reducing the capacity to pump blood out, which often leads to irregular heartbeat and heart failure. Cardiomyopathy is complex and heterogeneous heart disease, causes immense health and economic burdens worldwide. Despite the progress already made, prevention and control of cardiomyopathies are still challenging. There is still lagging continuous innovation in pharmaceuticals, medical devices, and diagnostics. It is necessary to explore further insights into molecular and pathological processes of the diseased heart to develop early diagnostic and new therapeutic strategies.

**Figure 1:**
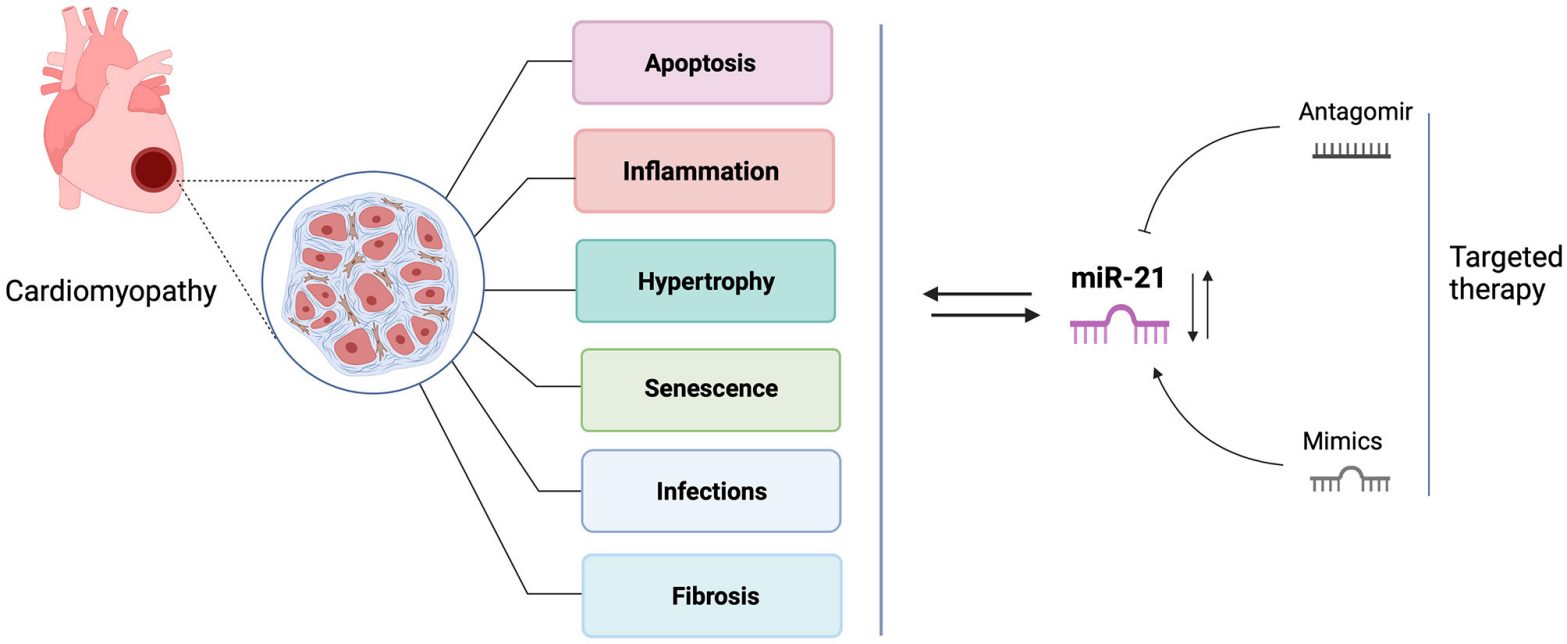
The roles of miR-21 in cardiomyopathies and targeted therapy. This figure is created with BioRender.com.

Over the past two decades, numerous research studies in biomarker identification and targeted therapy development have brought microRNA(miRNAs) research to the fore. miRNAs are short non-coding RNAs that repress target mRNAs expression by altering transcript stability or impacting mRNA translation (2). microRNAs play essential roles in the molecular network of cardiac development and cardiac disease pathogenesis (3). Of them, the most studied miRNA is miR-21, which is suggested as a predictive and diagnostic maker for many cardiac disorders, including heart failure, myocardial infarction, coronary artery disease, diabetic cardiomyopathy, and myocardial ischemic reperfusion injury (4–8). Yet its expression trends are inconsistent in different studies and vary depending on expression sites and disease progression (9). Cardiac stresses commonly induce miR-21 expression, mediating stress-related signaling pathways to protect cardiomyocytes from apoptosis. However, miRNA-21 up-regulation also results in myocardial fibrosis by activating cardiac fibroblasts to myofibroblasts and causing further damage to the heart (10). In addition, it was reported that miR-21 was not essential for pathological cardiac remodeling because miR-21 knockout and inhibition in mice both displayed cardiac hypertrophy and fibrosis in response to various cardiac stresses (11). These contradictory findings may indicate the multiple roles of miR-21 in cardiomyopathies, and the pathological activities may depend on the cell types and disease conditions. In this review, we present an update of current findings of miR-21 and further discussion on the contradictory role of miR-21 in the pathogenesis of cardiac diseases and its future therapeutic perspectives (Figure 1).

## Overview of miR-21

microRNAs (miRNAs), a family of short (~22 nucleotides) non-coding RNAs that negatively regulate gene expression by binding 3' untranslated region (3'UTR) of their target mRNAs, leading to translational repression and degradation (12). To date, over 2,000 miRNAs have been identified in humans since the first miRNA, lin-4 was discovered in Caenorhabditis elegans in 1993 (13). Of them, miR-21 has been gained the most interest of researchers, and the studies include a wide range of diseases covering cancer, metabolic diseases, and viral pathogenesis (14).

### Biogenesis

The human miR-21 gene has 3,433 nucleotides, located in chromosome 17q23.2, an intergenic region with the 3' UTR end of the Transmembrane Protein 49 (TMEM 49) (also known as Human Vacuole Membrane Protein 1, VMP-1) (15). miRNAs share a common biosynthetic pathway. The biogenic processing steps of miR-21 were first characterized in 2004 (16). miR-21 gene has two transcription sites, T1 (the minor transcription site) and T2 (the primary transcription site), and both are utilized for the initiation of transcription. RNA Pol II enzyme initiates miR-21 transcription and synthesizes full-length (~3.5-kb) primary miR-21 (pri-miR-21) in the nucleus (17). Following transcription, primary miR-21 requires two cleavage steps to become a mature and functional miRNA. First, pri-miR-21 is cleaved into ≈70-nt-long intermediate precursor miR-21(pre-miR-21) by the microprocessor complex that contains RNase III Drosha and a dsRNA binding protein DGCR8 (18, 19). Exportin 5 transports pre-miR-21 in a Ran-GTPase-dependent manner from nuclear to cytoplasm, where maturation can be completed (20). Upon export to the cytoplasm, a second RNase III enzyme, Dicer, removes the terminal loop from pre-miRNA and releases a small 22-nucleotide miRNA duplex. Following miRNA duplex loading, the precursor RNA-induced silencing complex (pre-RISC) quickly degrades the “passenger” strand to form a mature miRNA- RISC complex (21).

### Physiological Functions

It is shown that most human protein-coding genes contain one or more miRNA-binding sites, so that miRNAs strictly regulate protein expression (22). In miRNA- RISC complex, miRNAs serve as target recognition purpose. “miRNA seed” is a domain at 2–7 nucleotides position of the 5' end of miRNAs. It recognizes the 3' untranslated region (UTR) of mRNAs, and miRNAs bind to UTR with complementary base pairing fashion. Once locked on an mRNA, the RISC silencing complex promotes downregulation of the protein by repressing or degrading the mRNA (23).

### Targets Genes

miR-21 is proven to be involved in the pathogenesis of cardiovascular diseases through regulating downstream target genes. To date, several genes are identified as miR-21 targets in cardiomyopathy. The most recognized two target genes that involve in anti-apoptosis are programmed cell death 4 (PDCD4) (24) and phosphatase and tensin homolog deleted from chromosome 10 (PTEN) (25). In cardiac fibrosis, the primary three target genes are SMAD family member 7(Smad7) (26) and sprouty1/2 (SPRY1/2) (27, 28). Besides, some other targets are also suggested that contain miR-21 specific binding sites, including peroxisome proliferator-activated receptor alpha(PPAR-α) (29), a-kinase anchoring protein 8 (Akap8), and BRCA1 associated RING domain 1 (Bard1) (30), Jagged 1 (31), sorbin and SH3 domain-containing protein 2 (SORBS2) and PDZ and LIM domain 5 (PDLIM5) (32).

## Expression Patterns of miR-21 in Cardiomyopathy

The expression pattern of miR-21 in cardiovascular diseases has been extensively studied in recent years. Previous studies imply that there is a correlation between miR-21 expression level and cardiac diseases. Therefore, many studies were carried out to verify whether or not miR-21 can be a predictive or prognostic marker of heart diseases. Here we focus on the contradictory expression pattern of miR-21 in heart diseases (Table 1).

**Table 1:** miR-21 expression pattern in cardiomyopathies.

**Disease**	**Animal study**	**Clinical study**	**Biomarker**
MI	Rat Heart # (33) Mouse Heart* (34) Rat Heart↑ (31, 35) Mouse Heart↓ (36)	Plasma* (37) Serum ↑ (38)	✓
CAD	Mouse Plasma↑(39)	Plasma↑ (6, 39–41) PBMCs↓ (42)	✓
AF	Rabbit Heart↑ (43)	Left atria↑(44) Myocytes↑ (45) Plasma↓ (46) Serum ↓ (47)	✓
HF		Plasma ↑ (48) Serum ↓ (49) Plasma* (50)	✓
DCM	Mouse Heart ↑ (26, 51) Mouse Heart↓ (52)	Heart ↑ (53) Serum ↑ (54)	✓
I/R Injury	Mouse Heart ↑ (55, 56) Rat Heart ↓ (57)	Heart ↑ (58)	✓

Myocardial infraction (MI), Coronary artery disease (CAD), Atrial Fibrillation (AF), Heart Failure (HF), Diabetic cardiomyopathy (DCM), Ischemia-Reperfusion injury (I/R injury), Time-dependent change *, Site-dependent change ^#^, Increase ↑, Decrease ↓, Suggested as a biomarker ✓.

### Vitro Study and Animal Model Study

*In vitro* studies, two main cell types, cardiomyocytes and cardiac fibroblasts are used to illustrate the miR-21 expression pattern and function.

In most cases, miR-21 expression levels were elevated in stress conditions such as oxidative stress, high glucose, and hypoxia (59–62). However, oxygen-glucose deprivation (OGD) and palmitate exposure can considerably decrease miR-21 expression levels in cardiomyocytes (63, 64).

Furthermore, murine models were applied to study miR-21 expression profiles in cardiomyopathy showing different miR-21 responses to cardiac damage. miR-21 was down-regulated in infarcted areas but was upregulated in border areas in the acute myocardial infarction (AMI) rat model (33). In the myocardial infarction (MI) mouse model miR-21 expression changed in a time-dependent manner, increased in the first and second week but unchanged in the fourth week (34). Besides, two independent studies in mice models showed opposite miR-21 expression trends. One was observed an increase of miR-21 in the infarcted zone after MI, and the other showed a decrease of miR-21 after AMI (35, 36). Similarly, the reverse expression trends were also observed in ischemia/reperfusion (I/R) murine injury models. In the I/R injury mouse model, miR-21 was upregulated in the heart (55, 56), whilst heart miR-21 expression level decreased in the myocardial I/R injury rat model (57).

### Clinical Study

In the clinical studies, the expression level of miR-21 was measured in the blood and heart tissue of patients suffering from different heart diseases. The results showed divergent expression trends. In the case of atrial fibrillation (AF), it is suggested that miR-21 might be involved in the pathogenesis of atrial fibrosis due to its increased expression in left atrial tissue and positive correlation with atrial collagen content (44). Another study also showed that miR-21 was highly expressed in human atrial myocytes from patients with chronic atrial fibrillation compared to the sinus rhythm group (45). By contrast, different independent studies observed significantly decreased miR-21 levels in plasma or serum in AF patients (46, 47). Likewise, the reverse trends of miR-21 expression were also found in patients with coronary artery diseases. miR-21 expression levels were upregulated in plasma (6, 39–41), but dramatically decreased in peripheral blood mononuclear cells (PBMCs) (42).

In heart failure, for example, the upward trend expression of miR-21 was observed in circulation (48), but there was a decrease of miR-21 expression in serum in symptomatic heart failure patients (49). More surprisingly, patients with acute heart failure showed increased plasma miR-21 levels between admission and discharge of hospital, then decreased following clinical compensation period (50). Further, this time-dependent expression of circulating miR-21 occurred in patients with post-myocardial infarction as well. miR-21 decreased in the first 2 days and increased on the 5th day, then returned to the control level in the latter point of post-myocardial infarction (37). In another study, upregulated serum miR-21 expression was observed in elderly patients with acute myocardial infarction (AMI) (38).

In other cases, various cardiomyopathies, including dilated cardiomyopathy, left ventricular ejection fraction, and ischemic cardiomyopathy, upregulated miR-21 expression levels were shown in heart tissue and circulation (53, 54, 58, 65). In addition, increased miR-21 level was suggested as a new biomarker of cardiovascular disease-related premature death to predict patients at great risk of adverse outcomes (66, 67). However, unchanged expression miR-21 level was also reported in patients with hypertrophic cardiomyopathy (53).

In short, it has been shown that the distinct miR-21 expression pattern in one cardiac disease depending on different sample sources. Moreover, inconsistent trends were also observed from the same source of samples (such as plasma) in the same cardiac diseases.

## Functions of miR-21 in Cardiac Diseases

**Figure 2:**
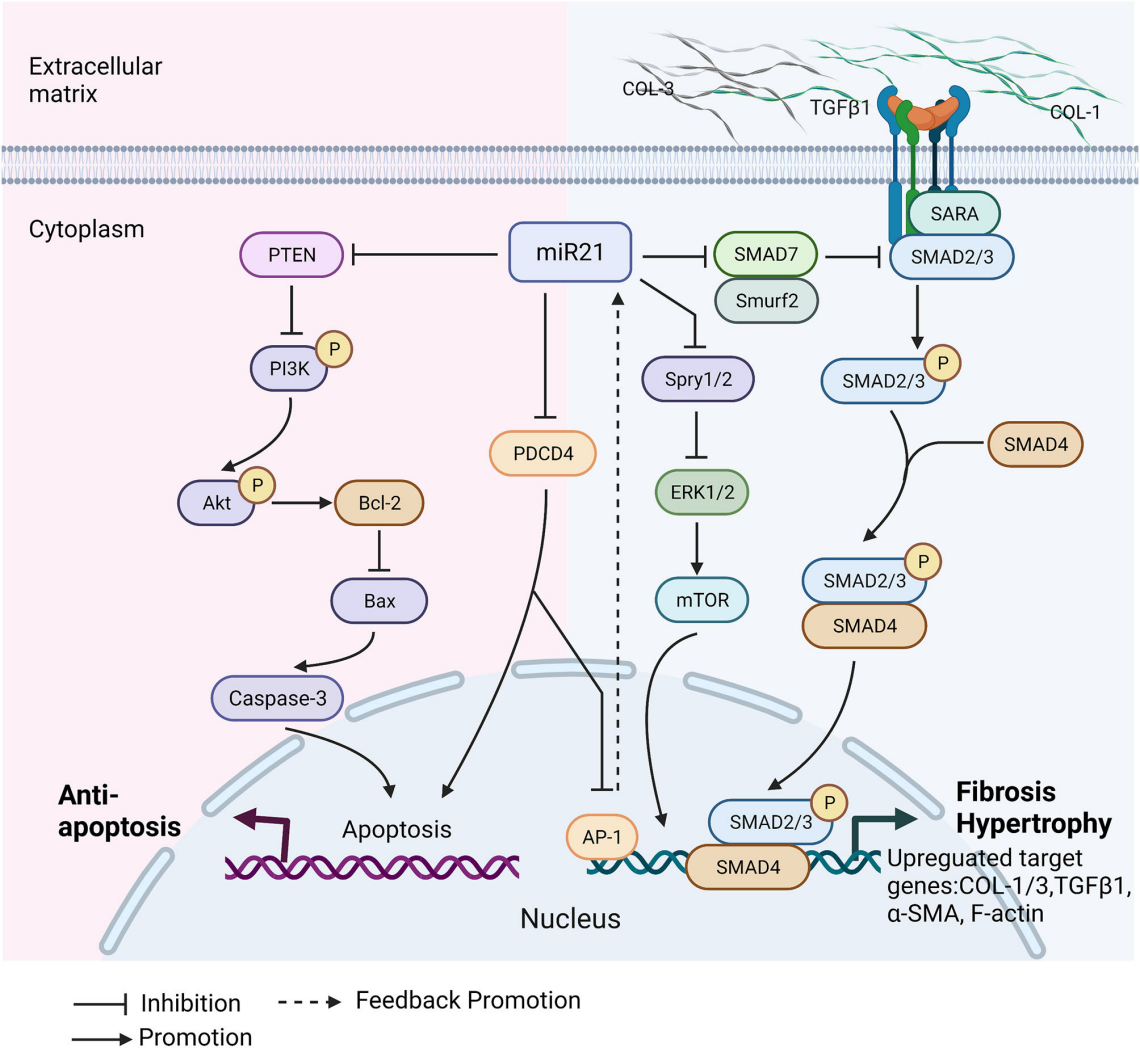
Molecular mechanisms of miR-21 involvement in cardiomyopathies. Cardiomyopathy alters miR-21 expression level in heart and circulation. miR-21 has protective effects on heart in cardiomyopathy. It regulates cardiac cell death by targeting PTEN and PDCD4. There is a positive promotion loop between miR-21 and AP-1. Overexpression of miR-21 inhibits PDCD4, and further increases AP-1, which is a transcription factor directly promotes miR-21 expression. PTEN downregulation activates PI3K/Akt pathway to promote cardiomyocytes survival and proliferation, that can protect against heart dysfunction. In the meantime, miR-21 has also negative effects on cardiac diseases' development. miR-21 regulates smad7/smad2/3 and Spry/ERK pathways to promote cardiac fibrosis by increasing collagens deposition, TGFβ1, α-smooth muscle actin (α-SMA) and filamentous actin (F-actin) polymerization. Spry/ERK/mTOR pathway contributes to myocardial hypertrophy through increasing myofibroblast survival. Collagen type I/III (COL1/3), PTEN, Phosphoinositide 3-kinases (PI3K), Protein kinase B(Akt), B-cell lymphoma 2 (Bcl-2), bcl-2-like protein 4 (Bax), Programmed Cell Death 4 (PDCD4), Activator protein 1(AP-1), Extracellular signal-regulated kinase (ERK), Sprouty 1/2(Spry 1/2), Mammalian target of rapamycin (mTOR), SMAD Specific E3 Ubiquitin Protein Ligase 2 (Smurf 2), Smad anchor for receptor activation (SARA), SMAD Family Member 2/3/4/7 (Smad2/3/4/7), Filamentous actin (F-actin), Alpha smooth muscle actin (α-SMA). This figure is created with BioRender.com.

There is no doubt that miR-21 is a crucial regulator of developing cardiac diseases and the healing process after heart injury. However, it plays an important but controversial role. For instance, overexpression of miR-21 reduced myocardial infarct size by 29% at 24 h and decreased the dimension of left ventricles at 2 weeks after acute myocardial infarction (AMI). At the same time, miR-21 rescued cardiomyocytes from ischemia-induced cell apoptosis by targeting programmed cell death 4 (PDCD4) and activator protein 1(AP1) (33). On the contrary, miR-21 expression increased in the myocardial infarction mouse model, and transfection of miR-21 mimics into cardiac fibroblasts (CFs) caused activation of cardiac fibroblasts promoted cardiac fibrosis (35). Here, we discuss the diverse functions of miR-21 (Table 2) and the underlying cellular and molecular mechanism in cardiomyopathy (Figure 2).

**Table 2:** miR-21 functions in cardiomyopathies.

**Disease conditions**	**Sample sources**	**Targets**	**Functions**	**References**
MI	Heart/CMs/MPs	PDCD4	Apoptosis ↓ Inflammation ↓	(33, 34, 36, 68)
HF	Heart	PTEN	Angiogenesis/CMs Survival ↑	(69)
DCM	Heart, CMs	Gelsolin	Hypertrophy ↓	(52)
I/R Injury	Heart, CMs	PDCD4/PTEN	Apoptosis ↓	(56, 70)
Valve Replacement	Heart		Apoptosis ↓	(65)
VMCs	Heart	MAP2K3	Necrosis/ viral titers ↓	(71)
HR	CMs	PTEN/PDCD4 Akap8/ Bard1	Apoptosis ↓	(30, 61, 70)
OGD/ H_2_O_2_/ Palmitate	NRCMs/ CPCs H9C2/ CSCs	PDCD4	Apoptosis ↓	(36, 60, 64, 70, 72)
MI	Heart/CFs	Smad7/Jagged1/ Spry1	Fibrosis ↑	(31, 35, 73)
HF	CFs	Spry1	Fibrosis ↑	(74)
DCM	Heart	SPRY1	Fibrosis ↑	(51)
I/R injury	CFs	PTEN	Fibrosis ↑	(55)
HHD	Heart	PDCD4	Fibrosis ↑	(75)
AF	Herat/CFs/CMs	Spry1/Smad7	Fibrosis/ arrhythmia ↑	(43–45)
CAD	Coronary artery		Plaque instability ↑	(41)
HG	CFs	DUSP8	Fibrosis ↑	(51, 59)
Ang-II	CPCs/NRCMs	PDCD4	Fibrosis/hypertrophy ↑	(62, 75)

*Myocardial infraction (MI), Viral myocarditis (VMC), Heart Failure (HF), Diabetic cardiomyopathy (DCM), Ischemia-Reperfusion injury (I/R injury), Hypoxia-Reoxygenation (HR), Oxygen-glucose Deprivation (OGD), Hydrogen peroxide (H_2_O_2_), Atrial Fibrillation (AF), Coronary artery disease (CAD), Hypertensive heart disease (HHD), High Glucose (HG), Angiotensin II (Ang II), Cardiac Fibroblasts (CFs), Cardiomyocytes (CMs), Macrophages (MPs), Differentiated Cardiac Progenitor Cells (CPCs), Primary Neonatal Rat Cardiac Myocytes (NRCMs), Rat Myoblasts (H9C2), Cardiac Stem Cells (CSCs), Programmed Cell Death 4 (PDCD4), Mitogen-activated Protein Kinase Kinase 3 (MAP2K3), Phosphatase and Tensin Homolog Deleted on Chromosome 10(PTEN), Sprouty Homologue 1 (Spry1), A-kinase Anchoring Protein 8 (Akap8), BRCA1 associated RING domain 1 (Bard1), Dual Specific Phosphatase 8 (DUSP8), SMAD Family Member 7 (Smad 7), Increase ↑, Decrease ↓*.

### Protective Roles

Positive functions of miR-21 were studied in a range of heart diseases by both gains-of-function and loss-of-function. In myocardial infarction (MI), infarct heart size was decreased, and heart function improved after transfection of miR-21 extracellular vesicles (EVs) or lentivirus overexpressing miR-21. The further mechanism study revealed that miR-21 downregulated programmed cell death 4 (PDCD4) and apoptosis markers in cardiomyocytes, including bcl-2-like protein 4 (bax) and caspase-3 (34, 36). The similar protective effects of miR-21 also showed in myocardial ischemia-reperfusion (IR), diabetic cardiomyopathy, and hypertrophic cardiomyopathy. miR-21 attenuates cardiac injury and promotes cardiac myocytes survival through targeting PDCD4, gelsolin, and well-known tumor suppressor gene PTEN (52, 56, 70). Apart from anti-apoptotic function, miR-21 plays an anti-inflammatory role in cardiomyopathy as well. miR-21 mimics notably decreased the rate of tumor necrosis factor (TNF)-α-induced apoptosis in human cardiomyocytes by activating the JNK/p38/caspase-3 signaling pathway (38). In the I/R injury study, the overexpression of miR-21 effectively inhibited the TLR4/NF-κB pathway that reduced the level of myocardial apoptosis and inhibited the release of pro-inflammatory factors (57). Furthermore, a study has presented a new targeted therapy that miR-21 nanoparticle delivery into cardiac macrophages could promote angiogenesis, reduce hypertrophy, fibrosis, and cell apoptosis in the remote myocardium (68). miR-21 also can promote the proliferation of cardiac stem cells (CSCs). miR-21 up-regulation with miR-21 mimics efficiently accelerated cell viability and proliferation in CSCs and protected them from oxidative injury by activating PTEN/PI3K/Akt pathway (72, 76). It is also proven that miR-21 can decrease cell apoptosis, inhibit viral progeny release in the heart, and protect it from coxsackievirus B3 infection through MAP2K3/P38 MAPK signaling pathway (77). Overall, miR-21 has beneficial effects on diseased cardiac tissues mainly by inhibiting PTEN and PDCD4 and promoting cell survival and proliferation.

### Detrimental Roles

#### Cardiac Fibrosis

Cardiac fibrosis is characterized by excessive and continuous collagens deposition and activation of cardiac fibroblasts and differentiation to myofibroblasts (69, 78). Various conditions can promote cardiac fibrosis, such as myocardial infarction, hypertension, and diabetic cardiomyopathy (79). Despite cardiac fibrosis is a self-healing process, it also leads to severe cardiac dysfunctions due to abnormal extracellular matrix (ECM) remodeling (80). In the significant number of studies, it has been demonstrated that miR-21 is involved in cardiac fibrosis (54, 75). miR-21 directly regulates downstream targets and engages in a variety of fibrotic activities that include increasing transforming growth factor β 1(TGFβ-1), cardiac fibroblasts activation, and endothelial-mesenchymal transition (EndMT) (26). Here, we summarize miR-21-mediated two main cardiac fibrosis signaling pathways in cardiomyopathies:

(i) miR-21/ TGF-β1/smad7 Pathway

Cardiac fibrosis is tightly dependent on transforming growth factor beta-1 (TGF-β1), contributing to cardiac fibroblast differentiation to myofibroblast (81, 82). In the diseased condition, miR-21 and TGF-β1 expression levels are commonly upregulated in cardiac fibroblasts. Functionally, there is a mutual promotion loop between miR-21 and TGF-β1: transfection of miR-21 mimics into cardiac fibroblasts increases TGF-β1 synthesis and secretion, and similarly, cardiac fibroblasts treat with TGF-β1 boost miR-21 expression (63). Studies suggest that Smad7 is an essential direct target gene of miR-21 and a critical negative regulator in this feedback loop between miR-21 and TGF-β1 (43). Smad7 is capable of inhibiting TGF-β1 signaling by preventing the phosphorylation of Smad2/3 or recruits the ubiquitin ligases Smurf1 and Smurf2 to induce proteasomal degradation of the receptor complexes (26, 83). miR-21 inhibition upregulated Smad7 expression, significantly alleviated myocardial fibrosis *in vivo*, and prevented cardiac fibroblasts activation *in vitro* study (35).

(ii) miR-21/ TGF-β1/spry1 Pathway

The Sprouty (Spry) family is first identified as a negative regulator of fibroblast growth factor (FGF) in Drosophila (73). Spry1 belongs to the Sprouty (Spry) family, an evolutionally conserved gene that acts as a potent inhibitor of the Ras/MEK/ERK pathway (84). Spry1 is a target of miR-21. The 3' untranslated region (UTR) of Spry1 mRNA contains several predicted microRNA-binding sites, of which only miR-21 levels are increased selectively in fibroblasts of the failing heart. Increased miR-21 blocks Spry1 and occurs further Inhibition of ERK–MAP kinase activity (74). This mechanism regulates fibroblast survival and growth factor secretion, TGF-β1 in particular. When TGF-β1 is over-expressed, that leads to downregulate the expression level of Spry1, thereby controlling the extent of interstitial fibrosis and cardiac hypertrophy (85). A study showed that miR-21 was highly expressed in left atria from patients with atrial fibrillation, which positively correlated with collagen content and caused a reduction of protein expression of Spry1 and increased connective tissue growth factor (CTGF), lysyl oxidase, and Rac1-GTPase (44). Likewise, another study demonstrated that miR-21 promoted post-myocardial infraction fibrosis by targeting Spry1, which inhibits ERK1/2 activation and mediates TGF-β/Smad2/3 pathway activation (73). Besides, overexpression of miR-21 induced myocardial fibrosis by regulating two other targets: Jagged 1 and dual specific phosphatase 8 (DUSP8). miR-21 suppressed DUSP8 expression and promoted high glucose-induced cardiac fibrosis through the JNK/SAPK and p38 signaling pathways (31, 59).

#### Cardiac Hypertrophy

In diabetic cardiomyopathy (DM), miR-21 knockout mice showed less severe cardiac hypertrophy and cardiac dysfunction than wide-type DM mice. Overexpression of miR-21 aggravated fibrosis, reduced autophagy through the miR-21/SPRY1/ERK/mTOR pathway (51).

#### Cardiac Senescence and Inflammaging

Inflammaging is a major risk factor for heart failure and overall cardiac disorders, but the underlying molecular mechanism remains unclear. It has been suggested that miR-21 is involved in cardiac senescence and inflammaging. Circulating miR-21 was higher in elderly patients with the cardiovascular disease than in the age-matched healthy control group, and miR-21 was correlated positively with inflammatory factors such as C-reactive protein and fibrinogen levels (86). In addition, another study showed that miR-21 was elevated in both aged myocardium and D-galactose-induced pseudo-aging mouse model, and further investigation showed that miR-21 promoted doxorubicin-induced cardiomyocyte senescence *in vitro* (87).

#### Viral or Bacterial Infection-Induced Myocarditis

miR-21 is one of the microRNAs that takes part in inflammatory pathogenesis in viral and bacterial myocarditis. Highly expressed miR-21-3p was observed in mouse heart challenged with lipopolysaccharide (LPS) and positively correlated with cardiac dysfunction. Meanwhile, the study suggested SORBS2 was a target gene of miR-21-3p (88). In Viral myocarditis, miR-21 was upregulated, and silencing miR-21 ameliorated viral myocarditis by blocking Th17 differentiation (71).

In brief, the conflicting data shows contradictory functions of miR-21 in cardiomyopathies. Indeed, the tasks of miR-21 *in vivo* studies are mysterious. Every research is unique, studying different cardiac diseases, even though looking at the same condition still differs in animal models or the progression time-point of diseases. It is hard to find identical repeated studies to make the comparison among results.

However, the results of Vitro studies show that high consistency depends on specific cell types. If the studied subject is cardiomyocytes, the results indicate miR-21 has a protective effect on the heart, such as anti-apoptotic effect on cardiomyocytes; if the research subject is changed to cardiac fibroblasts, the results lead to demonstrate a negative role of miR-21 in myocardial fibrosis. Intriguingly, neither Vivo studies nor Vitro studies try to illustrate both effects of miR-21 at the same time in one cardiac disease. Therefore, delicate and sophisticated research studies require to show miR-21 distinct functions in different cell types *in vivo*.

## Clinical Implications of miR-21

### Biomarker

miR-21 has been proposed as a biomarker in a variety of heart diseases. A clinical biomarker is defined as “an indicator of normal biological processes, pathogenesis, or responses to an exposure or therapeutic intervention” (89). For instance, troponin I (cTnI) and T (cTnT) are unique heart proteins and are specific and sensitive biomarkers of myocardial injury in various cardiovascular disorders (90). Ideal biomarkers have the potential to provide valuable information that aids the diagnosis of a disease and predicts disease progression and outcomes. Bradford Hill's Guidelines can be applied to establish and evaluate pre-biomarkers (91). Here, we list the first three criteria to show whether or not miR-21 can be a biomarker of cardiomyopathies.

The first essential requirement is that there should be strong associations between a biomarker and the outcome of a clinical disorder. Undoubtedly, miR-21 regulates key pathways and tightly relates to cardiac pathogenesis, such as cardiac fibrosis. Expression changes of miR-21 were observed in many studies, and the pathological roles were demonstrated by both gain- and loss- of-function study.

Additionally, consistent findings should be observed by different individuals in different places with different samples. miR-21 is not fulfilled with this point. The inconsistent expression levels and contradictory functions have been reported throughout the last two decades. miR-21 expression levels in circulation and heart tissue were various in independent studies. A plausible reason is that there are no standard reference values for miR-21 expression in physiological and pathological conditions. The expression levels in control groups might vary from subject to subject.

Most importantly, an ideal marker is supposed to express at a specific site and associate with a particular disease. miR-21 is not a specific miRNA in the heart. miR-21 is expressed abundantly and ubiquitously in some other multiple types of tissues, such as the liver, kidney, nervous system, and immune cells (92). Not only does diseased heart tissue contribute to the circulation level of miR-21, but other tissues, especially those globally involved in various diseases, namely immune cells, also secret miR-21 to respond to pathological stimuli *via* inflammation pathways (93). In addition, miR-21 is not a disease-specific marker either. miR-21 is suggested as a marker in various cardiovascular diseases, including heart failure, atrial fibrillation, and dilated cardiomyopathy. Apart from cardiovascular diseases, numerous studies implicated miR-21 as a biomarker in many other diseases ranging from cancer, renal fibrosis, and diabetes (94). So far, miR-21 cannot be considered an ideal biomarker for a specific cardiomyopathy.

### Targeted Therapy

With increasing interest in miRNAs, miRNA-based drug development is already in progress. Over the last decade, several biopharmaceutical industries have launched miRNA projects in their development pipeline. For example, the first miRNA-targeted drug for treating hepatitis C virus (HCV) infection sponsored by Santaris Pharma in 2012 is miravirsen. It is an LNA-modified antimiR oligonucleotide, targets miR122 (95). miRNA-based drugs have two main types: AntagomiRs, which can specifically bind to target miRNAs in a complementary fashion and inhibit their functions; another is synthetic miRNA mimics, which can restore the reduced expression of miRNAs in disease conditions (96). Currently, there are going on many miR-21-based diagnostics and therapeutic clinical trials in a range of diseases, such as Predictive and Prognostic Value of Inflammatory Markers in Stage IV Colorectal Cancer (NCT04149613), miR-200b & miR-21 in Diabetic Wounds recruiting (NCT02581098), and Study of Lademirsen (SAR339375) in Patients with Alport Syndrome (HERA) (NCT02855268). Of note, miRNA-based drugs are currently not available for diagnostics and therapeutic purposes.

Similarly, miR-21 has emerged as a potential therapeutic target in cardiomyopathy due to its participation in the pathogenesis of many cardiac diseases *via* regulating cardiomyocytes apoptosis and cardiac fibrosis. Numerous preclinical studies have been carried out to illustrate its therapeutic outcomes in cardiomyopathies. For instance, a study demonstrated that miR-21 overexpression by nucleofection in human cardiac fibroblasts with the expression vector leads to upregulation of collagen expression. In contrast, miR-21 Inhibition by LNA anti-miR-21 reduced collagen expression and alleviated cardiac fibrosis (82).

However, developing miR-21 targeted treatment for cardiomyopathies has several challenges. Firstly, It is challenging to apply repression or restoration, because the confusion of miR-21 expression pattern in cardiomyopathies. Besides, it is problematic for targeted therapy that miR-21 is not an organ or tissue-specific miRNA, not even cell type-specific miRNA. Hence, issues with off-target effects and undesired on-target effects have to be taken into account (97).

Despite the considerable understanding of miR-21 regulation, further studies require to obtain sufficient knowledge of miR-21 in its spatial and temporal regulation. miR-21 expression is tightly regulated by multiple factors. Several promotors can effectively induce miR-21 expression at the transcriptional level, including AP-1, STAT3, Ras, ERK1/2, and EGFR (98–102). Oppositely, some transcriptional suppressors have also been reported. For instance, miR-21 transcription was repressed by NFI, C/EBP, Gfi1, and estrogen receptor (98, 103, 104). Moreover, miR-21 expression is regulated at the post-translational level. TGFβ and BMP4 (a member of the TGFβ superfamily) upregulate pre-mir-21 expression and SMADs (SMAD1/5 and SMAD2/3) mediate pri-mir-21 expression (105). On the contrary, BMP6 (a member of the TGFβ superfamily) has been shown to inhibit miR-21 expression (106). In addition, miR-21 expression can be modulated by other non-coding RNA associated with various molecular pathways. Tumor-Associated Long ncRNA Expressed on Chromosome 2(TALNEC2) was highly expressed in myocardial ischemic patients. Overexpression of TALNEC2 downregulated miR-21 and aggravated hypoxic injury by miR-21/PDCD4-mediated activation of the Wnt/β-catenin signaling pathway in myocardial ischemic injury (107). In cardiovascular diseases, LncRNA MEG3 is downregulated and negatively correlates with miR-21 levels. MEG3 overexpression suppresses endothelial cell proliferation and migration and reduces proteoglycan, type I, and V collagen expression through the decrease of miR-21 (108).

## Summary and Perspective

The heart is an important and complex organ. Many diseases can affect the functionality of the heart to cause a range of complex conditions. Cardiomyopathy is defined as a heart muscle disease, but not only cardiomyocytes and some other heart cells are also involved in the occurrence and progression of cardiomyopathy. The cardiac muscle is composed of various cell types. Cardiomyocytes account for 25–35% of all cells in the adult cardiac muscle, and other heart cell types make up 65–75%, including endothelial cells, smooth muscle cells, fibroblasts, and immune cells (109). Both cardiomyocytes and non-cardiomyocytes respond to physiological and pathological stress, such as cardiomyocytes apoptosis, cardiac fibroblast activation, and immune cell infiltration. Those all play indispensable roles in heart pathogenesis (110). miRNAs expression profiles may be completely distinct based on different heart cell types (111, 112). Similarly, miR-21 may vary in expression signatures and functions in different heart cells. As reported, miR-21 overexpression activates fibroblast to trigger the fibrosis process but stimulates proliferation of cardiomyocytes against stress overloaded conditions. The detailed expression map of miR-21 remains elusive, so that it is challenging to apply miR-21 as a biomarker in heart disease diagnosis or prognosis. Moreover, the biology of miR-21 has not been fully clarified. It is not clear that the regulation details in miR-21 biogenesis, secretion, and degradation. Thus, further studies are necessary to understand the molecular basis of distinct functions and identify specific target genes of miR-21 in different tissues. Furthermore, it is not only miR-21, the multiple miRNAs involved in heart diseases. It has been reported that dozens of miRNAs are associated with heart failure, myocardial infarction, and arrhythmia (113). Studies show that miR-1, miR133a, miR-208a/b, and miR-499 have explicitly enriched the myocardium and might be specific therapeutic targets in cardiomyopathy (114). For example, miR-499 participates in the development of myocardial hypertrophy and fibrosis by targeting many intracellular signaling molecules and transcription factors, including Akt, mitogen-activated protein kinases (MAPKs), and early growth response 1/2 (Egr1/2) (115). Besides, miR-21 may interact with other microRNAs that participate in cardiac pathogenesis. A cancer study demonstrated a high level of miR-21 increased miR-499 expression level by stabilizing mature miR-499 and affecting its turnover rate, resulting in the suppression of PDCD4 in head and neck squamous cell carcinoma (HNSCC) (116).

Nonetheless, miR-21 studies shed light on the understanding of cellular and molecular mechanisms of cardiomyopathy and offered a potential therapeutic target for clinical intervention.

## Author Contributions

S, GP, and MB: concept and design. S, RF, and LS: drafting of the manuscript. All authors have read and agreed to the published version of the manuscript.

## Funding

This review was supported by the Ministero dell'Istruzione, dell'Università e della Ricerca Scientifica (Grants PRIN 2017).

## Conflict of Interest

The authors declare that the research was conducted in the absence of any commercial or financial relationships that could be construed as a potential conflict of interest.

## Publisher's Note

All claims expressed in this article are solely those of the authors and do not necessarily represent those of their affiliated organizations, or those of the publisher, the editors and the reviewers. Any product that may be evaluated in this article, or claim that may be made by its manufacturer, is not guaranteed or endorsed by the publisher.
