# Corrigendum: miR-21 in Human Cardiomyopathies

**DOI:** 10.3389/fcvm.2022.913429

**Published:** 2022-04-25

**Authors:** Surina, Rosaria Anna Fontanella, Lucia Scisciola, Raffaele Marfella, Giuseppe Paolisso, Michelangela Barbieri

**Affiliations:** ^1^Department of Advanced Medical and Surgical Sciences, University of Campania “Luigi Vanvitelli”, Naples, Italy; ^2^Mediterrannea Cardiocentro, Napoli, Italy

**Keywords:** miR-21, cardiomyopathies, biomarkers, targeted therapy, fibrosis

An author name was incorrectly spelled as “Surina Surina.” The correct spelling is “Surina.”

The authors apologize for this error and state that this does not change the scientific conclusions of the article in any way. The original article has been updated.

## Publisher's Note

All claims expressed in this article are solely those of the authors and do not necessarily represent those of their affiliated organizations, or those of the publisher, the editors and the reviewers. Any product that may be evaluated in this article, or claim that may be made by its manufacturer, is not guaranteed or endorsed by the publisher.

